# Editorial: Metabolomics in Crop Research—Current and Emerging Methodologies

**DOI:** 10.3389/fpls.2019.01013

**Published:** 2019-08-02

**Authors:** Marta Sousa Silva, Carlos Cordeiro, Ute Roessner, Andreia Figueiredo

**Affiliations:** ^1^Laboratório de FTICR e Espectrometria de Massa Estrutural, Faculdade de Ciências, Universidade de Lisboa, Lisbon, Portugal; ^2^Centro de Química e Bioquímica, Faculdade de Ciências, Universidade de Lisboa, Lisbon, Portugal; ^3^School of BioSciences, The University of Melbourne, Melbourne, VIC, Australia; ^4^Biosystems and Integrative Sciences Institute, Faculdade de Ciências, Universidade de Lisboa, Lisbon, Portugal

**Keywords:** metabolomics, mass spectrometry, nuclear magnetic resonance, metabolic profiling, crop development

The plant metabolome is highly complex, being composed of over 200,000 metabolites (Fiehn, 2002). The characterization of these small molecules has been crucial to study plant growth and development as well as their response to environmental changes. The potential of metabolomics in plant research, particularly if applied to crop plants, is immense. Besides the aforementioned applications, it is extremely valuable in the discovery of biomarkers and in the improvement of crop yield and quality (Alseekh et al., 2018). This Frontiers Research Topic addresses many applications of metabolomics to crop research, based on different analytical platforms, including mass spectrometry, and nuclear magnetic resonance (NMR). It comprises 13 articles from 109 authors that show the importance and the contribution of metabolomics in the analysis of crop's traceability and genetic variation, in the study of fruit development, and in the understanding of the plant's response to the environment and to different biotic and abiotic stresses.

Numerous agricultural and food products are recognized by their qualities that result mainly from their geographical origin (Vandecandelaere et al., 2009). An unequivocal traceability of a crop guarantees, not only its origin, but also its quality and safety. Busconi et al. followed a targeted metabolomics approach to specifically analyse the phenolic profile of *Vanilla* × *tahitensis* and its relation to plant traceability. The phenolic compound profile of different *Vanilla* × *tahitensis* clearly discriminated the Papua New Guinea samples from the Tahitian ones from French Polynesia. Additionally, it was possible to separate the *Vanilla* cultivars that comprised the two Tahitian samples: one exclusively from “Haapape” cultivar and the other from a mixture of both “Haapape” and “Tahiti” cultivars. Within the same region, latitude, and altitude also influence the metabolic composition of a plant. Demasi et al. analyzed different populations of *Lavandula angustifolia* (lavender) grown in nine peripheral alpine regions for future selection of higher quality flowers and essential oils. A targeted strategy based on the analysis of volatile compounds and essential oils revealed that latitude significatively influenced phytochemical composition, while altitude didn't affect the phytochemical profile of *L. angustifolia*. These results can be explored for the future ranking of lavender cultivation sites, thus promoting quality and value linked to geographical origin.

In the search for quality traits within crops, untargeted metabolomics approaches have been also very useful. Ellis et al. performed untargeted metabolic profiling of *Pisum sativum* (pea) mature seeds from genetically marked lines to identify which genotypes were enriched in specific compounds related to end-use quality traits. Indeed, there were sets of compounds in mature seeds associated with their genetic variation, and this information can be used to assist future breeding programmes. Seed quality can be compromised by the occurrence of pests and diseases in the crop. *Pisum sativum* plants can be affected by the pea aphid *Acyrthosiphon pisum*, a phloem-feeding insect comprised of different biotypes (or host races), each specialized on a specific crop legume species, including *Medicago sativa* (alfalfa) and *Trifolium pratense* (red clover), besides *P. sativum* (Peccoud et al., 2009). Interestingly, all the host races of this insect can develop on *Vicia faba* (faba bean). Sanchez-Arcos et al. studied these four plant-herbivorous insect systems using an untargeted metabolomics strategy to identify the metabolites involved in the specificity of pea aphid interaction with the different host plants. All these crop legumes are also affected by other pathogens, including the *Aphanomyces euteiches*, a soil-borne oomycete that causes Aphanomyces root rot (ARR) (Gaulin et al., 2007). Bazghaleh et al. followed a targeted metabolomics approach focused on polyphenolic profiling, to study the resistance of several lentil genotypes (*Lens* sp.) to *A. euteiches* infection. Several differences were found within the root polyphenolic profiles from the wild and the different cultivars of lentils, and between healthy and infected roots. The observed relationship between polyphenol composition and tolerance to *A. euteiches* will be valuable for future selection of resistant plants. Similar results were obtained by Bernardi et al. while studying the susceptibility of *Zea mays* (maize) cultivars to the fungus *Fusarium verticillioides*. Again, a targeted strategy was chosen, focusing on the screening of phenolic compounds from pigmented and non-pigmented maize cultivars. The maize cultivar with highest phenolic content showed highest resistance to *Fusarium* infection, a promising result toward the selection of more resilient maize plants.

Studying another plant-pathogen system, Chitarrini et al.'s work also showed a great potential for future application for the development of resistant varieties. Using different analytical methods, they identified biomarkers in a resistant grapevine associated with the defense against the biotrophic oomycete *Plasmopara viticola*, the causative agent of downy mildew. This study contributes for a better understanding of the mechanisms of grapevine interaction and resistance to downy mildew. Negrel et al. took a further step in elucidating this grapevine-*P. viticola* interaction and characterized *P. viticola*'s metabolome using an untargeted metabolomics approach. These pathogen biomarkers can be used to in the development of a monitoring assay for the early detection of *P. viticola* in grapevine.

While analyzing both compatible and incompatible interactions between tomato (*Solanum lycopersicum*) and *Pseudomonas syringae*, López-Gresa et al. characterized the profile of volatile organic compounds associated with the tomato immune response to this bacteria. These results can be used in the future development of resistant tomato plants, thus preventing this agricultural problem and contributing to a more sustainable production. Another concern regarding stable fruit development is the availability of an optimal light environment. This has been a problem in several countries with fewer daylight hours. For tomato, a supplementary light system is often used in plants grown in greenhouses. However, an adequate plant growth and fruit development depends on the correct implementation of lightning strategies. Fukushima et al. performed an integrative omics strategy to investigate the metabolic changes in early fruit development of single-leaf tomato plants, with only one fruit truss, exposed to different intensities of red LED (light-emitting diode) light. The compounds that responded to the LED treatment and most contributed to the increase of fruit size of tomato plants were metabolites mainly involved in carbohydrate metabolism and the biosynthesis of several amino acids. This is quite relevant given the importance of carbon allocation for fruits during their development, for which a balanced source-sink relationship is essential to ensure adequate fruit nutritional quality and yield (Smith et al., 2018). Following this line of research, Beshir et al. used isotopically labeled substrates and metabolomics to investigate carbon re-allocation changes throughout the development of apple fruit. For the first time it was possible to create a thorough understanding of the metabolic changes' dynamics occurring during the different growth stages of fruit development using dynamic isotope labeling experiments.

Productivity increase in a crop and the efficient use of resources has been achieved through controlled growth in plant factories. Although not a natural environment, these closed production systems, with controlled lightning strategies and reduced environmental pollutants, became more sustainable and attractive to the food industry (Kosai, 2013). Tamura et al. analyzed the metabolite profiles of lettuce leaves grown under hydroponic conditions or fertilized soil, to investigate how cultivation conditions affected leaf metabolic composition. The results showed that the metabolic profile of both lettuce cultivars analyzed was greatly influenced by the cultivation method. Among the affected metabolites are the ones responsible for taste and functional ingredients, like amino acids, and phenolic compounds. Neugart et al. also analyzed the effect of soil fertilization with biological waste compost in the metabolic composition of *Brassica rapa* ssp. *Chinensis* (pak choi) sprouts. Indeed, the addition of biological waste from food production (coffee, aronia, and hop) strongly affected sprout metabolic profile, by increasing the concentration of carotenoids and decreasing the glucosinolates and phenolic compounds. The studies from Tamura et al., Fukushima et al., and Neugart et al. show that a detailed assessment of the effect of alternative cultivation systems, like greenhouses, and plant factories, is crucial in the quality and nutritional value of crop products, particularly the evaluation of the light, soil, and fertilization conditions.

Metabolomics has a huge potential in crop plant research. This Research Topic presents current and emerging approaches in metabolomics applied to crop research that we believe will be a future reference in the field and contribute to improve plant productivity and quality. The currently available analytical platforms provide different strategies for metabolomics studies, from targeted, quantitative metabolite profiling, to global untargeted metabolic fingerprinting. In the field of quantitative metabolomics analysis, NMR and mass spectrometry coupled to liquid chromatography (LC-MS) or gas chromatography (GC-MS) are the techniques of choice (Lei et al., 2011). However, currently, Fourier-Transform Ion-Cyclotron-Resonance mass spectrometry (FT-ICR-MS) offers the greatest potential for untargeted metabolomics, being able to simultaneously detect and identify thousands of metabolites in very high-throughput assays, providing extreme-resolution and ultra-high-mass accuracy, and successfully used in crop metabolomics (Aliferis and Jabaji, 2012; Adrian et al., 2017; Maia et al., 2019).

## Author Contributions

This manuscript was written by MS and AF. It was revised and edited by all authors prior to submission.

### Conflict of Interest Statement

The authors declare that the research was conducted in the absence of any commercial or financial relationships that could be construed as a potential conflict of interest.
